# Construction of a Ferroptosis-Related Long Non-coding RNA Prognostic Signature and Competing Endogenous RNA Network in Lung Adenocarcinoma

**DOI:** 10.3389/fcell.2021.751490

**Published:** 2021-11-08

**Authors:** Xiang Fei, Congli Hu, Xinyu Wang, Chaojing Lu, Hezhong Chen, Bin Sun, Chunguang Li

**Affiliations:** ^1^Department of Thoracic Surgery, Changhai Hospital, Navy Military Medical University, Shanghai, China; ^2^Department of Medical Oncology, Shanghai Pulmonary Hospital, Thoracic Cancer Institute, Tongji University School of Medicine, Shanghai, China; ^3^Department of Thoracic Surgery, Renji Hospital, Shanghai Jiao Tong University School of Medicine, Shanghai, China; ^4^Department of Molecular Oncology, Eastern Hepatobiliary Surgical Hospital & National Center for Liver Cancer, Navy Military Medical University, Shanghai, China

**Keywords:** LUAD, ferroptosis, lncRNAs, prognostic signature, ceRNA network

## Abstract

Ferroptosis-related genes play an important role in the progression of lung adenocarcinoma (LUAD). However, the potential function of ferroptosis-related lncRNAs in LUAD has not been fully elucidated. Thus, to explore the potential role of ferroptosis-related lncRNAs in LUAD, the transcriptome RNA-seq data and corresponding clinical data of LUAD were downloaded from the TCGA dataset. Pearson correlation was used to mine ferroptosis-related lncRNAs. Differential expression and univariate Cox analysis were performed to screen prognosis related lncRNAs. A ferroptosis-related lncRNA prognostic signature (FLPS), which included six ferroptosis-related lncRNAs, was constructed by the least absolute shrinkage and selection operator (LASSO) Cox regression. Patients were divided into a high risk-score group and low risk-score group by the median risk score. Receiver operating characteristic (ROC) curves, principal component analysis (PCA), and univariate and multivariate Cox regression were performed to confirm the validity of FLPS. Enrichment analysis showed that the biological processes, pathways and markers associated with malignant tumors were more common in high-risk subgroups. There were significant differences in immune microenvironment and immune cells between high- and low-risk groups. Then, a nomogram was constructed. We further investigated the relationship between six ferroptosis-related lncRNAs and tumor microenvironment and tumor stemness. A competing endogenous RNA (ceRNA) network was established based on the six ferroptosis-related lncRNAs. Finally, we detected the expression levels of ferroptosis-related lncRNAs in clinical samples through quantitative real-time polymerase chain reaction assay (qRT-PCR). In conclusion, we identified the prognostic ferroptosis-related lncRNAs in LUAD and constructed a prognostic signature which provided a new strategy for the evaluation and prediction of prognosis in LUAD.

## Introduction

Lung cancer is the disease with the highest morbidity and mortality, among which lung adenocarcinoma (LUAD) is accounted for 40–50% of all lung cancer cases (Bray et al., 2018). At present, local resection is still the first choice for the treatment of LUAD. In recent years, molecular targeted therapy and immunotherapy have developed rapidly, which has brought success in the clinical treatment of a series of malignant tumors. Although the prognosis of some patients has improved significantly with the application of targeted therapy and immunotherapy, the survival rate is far from satisfaction (Miller et al., 2019). There is evidence showing that identification and application of novel biomarkers could bring benefits for the effective treatment of patients (Xu et al., 2021).

Ferroptosis is a form of regulatory cell death with iron dependence, caused by excessive lipid peroxidation and related to the occurrence of a variety of tumors and therapeutic response (Chen et al., 2021). Ferroptosis also plays an important role in LUAD. CAMP responsive element binding protein 1 (CREB) can directly bind to the promoter region of GPX4 to promote its expression, thereby inhibiting potential ferroptosis and promote the growth of LUAD (Wang Z. et al., 2021). P53 is a well-known tumor suppressor, which mediates apoptosis and cell-cycle arrest. In addition, it also can modulate ferroptosis in an ALOX12-dependent way and inhibit the proliferation of H1299 (Chu et al., 2019).

The abnormal expression of lncRNAs in LUAD is widely involved in the process of tumor proliferation and metastasis (Li and Chen, 2016). PD-L1-lnc was reported to directly bind to c-Myc and enhance its transcriptional activity, ultimately promoting proliferation and invasion of LUAD (Qu et al., 2021). Knockdown of lncRNA MSC-AS1 could inhibit LUAD cell growth and accelerate apoptosis through miR-33b-5p/GPAM axis (Li S. et al., 2021). What’s more, accumulating studies demonstrated that lncRNAs also regulated ferroptosis of lung cancer by interacting with ELAVL1 or miR-365a-3p (Wang et al., 2019; Gai et al., 2020). Previous studies have shown that ferroptosis-related lncRNAs could be prognostic risk factors and the signatures constructed with them can be used to distinguish high-risk patients from low-risk patients (Cai et al., 2021; Tang et al., 2021). However, the full role of ferroptosis-related lncRNAs in LUAD needs to be further explored.

Therefore, we retrieved ferroptosis-related genes from previous studies and mined potential dysregulated ferroptosis-related lncRNAs through bioinformatic analysis, based on the LUAD dataset of TCGA. Our study showed 33 lncRNAs were differentially expressed in LUAD and correlated with prognosis. Furthermore, a ferroptosis-related lncRNA prognostic signature (FLPS) was constructed based on LASSO regression. We confirmed the validity of FLPS through ROC, PCA, and Cox regression. Finally, a nomogram was constructed to predict overall survival (OS) of LUAD patients and a ceRNA network was built to further clarify the function of those lncRNAs in LUAD.

## Materials and Methods

### Data Collection

The RNA-seq transcriptome data, including 497 LUAD samples and 54 adjacent normal tissues, and corresponding clinical data were extracted from TCGA database^1^ for differential expression analysis. All patients included in the prognostic analysis fit the following criteria: (1) histologically confirmed LUAD and (2) available information on gene expression and survival. Lastly, 468 patients with corresponding clinicopathological information were enrolled for further study. A total of 468 patients with LUAD were randomly classified into a training cohort (235 patients) and a test cohort (233 patients) at a 1:1 ratio by using the “caret” package, according to previous studies (Yang et al., 2019; Geng et al., 2021). Then 283 ferroptosis-related genes were retrieved from the previous study, including 132 drivers and 151 suppressors (Liu et al., 2020). TCGA pan-cancer data, including RNA-seq and clinical data and stemness scores based on mRNA (RNAss) and DNA-methylation (DNAss) of LUAD were extracted from xena browser^2^. TCGA pan-cancer data include 33 cancer types. Among them, 18 cancer types had more than five associated normal tissue samples and were used to be further investigated.

### Establishment and Validation of the Ferroptosis-Related Long Non-coding RNA Prognostic Signature

The annotation of lncRNA was performed as a previous study (Tu et al., 2020). Pearson correlation was used to mine ferroptosis-related lncRNAs (with the | Person R| > 0.4 and *p* < 0.001).

Differentially expressed genes (DEGs), miRNAs (DEMs) and lncRNAs between tumor and adjacent normal tissues were identified by using the “limma” or “edgeR” R package (| log2FC| > 1, FDR < 0.05). Univariate Cox analysis of overall survival (OS) was performed to screen ferroptosis-related genes and lncRNAs with prognostic value. Then, R package “glmnet” was used to conduct least absolute shrinkage and selection operator (LASSO) Cox regression. A ferroptosis-related lncRNA prognostic signature (FLPS) was constructed for the LUAD patients, which involved six ferroptosis-related lncRNAs. The risk score was calculated as the formula:


$$\mathrm{Risk}\mathrm{score}=\sum_{i=1}^{n}\beta i\chi i$$

where β*i*means the coefficients, whereas χ*i* is the FPKM value of each ferroptosis-related lncRNAs.

Risk scores were calculated for all patients in our study. Kaplan–Meier curve was used to compare the OS between the high-risk and low-risk group through R package “survival” and “survminer.” Time-dependent ROC analyses and the area under the curve (AUC) were performed to assess the model by using “timeROC.” Principal component analysis (PCA) and scatter diagrams were performed by R package “ggplot2.”

### Functional Enrichment and Immune-Related Scores Analysis

Differentially expressed genes between the high-risk subgroup and low-risk group were identified based on the standards of | log2FC| > 1 and *p* < 0.05 using the R package “limma.” Then the DEGs were inputted into the “Metascape” website for functional and pathway enrichment analysis (Zhou et al., 2019). In addition, GSEA software was used to explore the hallmarks which were highly enriched in the high-risk group. The infiltrating score of 16 immune cells and the activity of 13 immune-related pathways were calculated with single-sample gene set enrichment analysis (ssGSEA) in the “gsva” R package. The immune, stromal and estimate score for each patient was calculated by the R “estimate” package.

### Competing Endogenous RNA Network Construction

The online tool LncBase V2.0^3^ (Paraskevopoulou et al., 2016) was used to predict the downstream target miRNA with a 0.6 threshold (Paraskevopoulou et al., 2016). TargetScan^4^ (Agarwal et al., 2015), miRDB^5^ (Chen and Wang, 2020), and miRWalk^6^ (Dweep and Gretz, 2015) were used to predict the target genes. Then, the predicted target genes and miRNAs were intersected with the DEGs and DEMs, respectively. MiRNAs or mRNAs that were not consistent with the expression of lncRNAs or miRNAs were eliminated. Then the network of lncRNAs, miRNAs, and mRNAs was constructed by Cytoscape (version 3.8.2) (Shannon et al., 2003).

### Screening of Hub Genes and Survival-Related Genes

The STRING database^7^ (Szklarczyk et al., 2021) was applied to construct PPI network and discover the relationship among the target genes with high confidence (0.7). Then, the gene relationship was imported into Cytoscape and the top 10 hub genes were calculated by cytoHubba. Kaplan–Meier curve and log-rank test were used to mine prognostic genes.

### Samples and Quantitative Real-Time Polymerase Chain Reaction

A total of 20 paired LUAD tissues and corresponding adjacent non-tumorous tissues were obtained from patients who underwent radical resection of lung cancer in Changhai Hospital, from June 2020 to September 2020. This study was approved by Ethics Committee of Changhai Hospital (Shanghai, China), and informed consent was signed before the operation for each patient. Total RNA was extracted from samples with TRIzol^TM^ Reagent (Invitrogen). RNA was reverse-transcribed using PrimeScript^TM^ RT Master Mix (Takara). Real-time PCR was performed with TB Green^TM^ Premix EX Taq^TM^ II (Takara). The expression levels of lncRNAs were normalized by GAPDH. The sequences of primers were as follows: AC021016.1 forward 5′-GGGTCAAGCACACTGAGGGT-3′, and reverse 5′-ACCAGGTGTGAACCCTTGGG-3′; KTN1-AS1 forward 5′- AGGGAAATTTGGGCAGAAGT-3′, and reverse 5′-GTTACCC GTGTGAGCCTGAT-3′; GAPDH forward 5′GTCTCCTCT GACTTCAACAGCG-3′, and reverse 5′-ACCACCCTGTTGCT GTAGCCAA-3′.

### Statistical Analysis

All statistical analyses were performed using R language 4.0.4 version and attached packages. Differentially expressed genes (DEGs), miRNAs (DEMs), and lncRNAs between tumor and adjacent normal tissues were identified by Wilcox test. Kaplan–Meier curve and log-rank test were used to compare OS between subgroups. Time-dependent ROC analyses and the area under the curve (AUC) were used to assess the performance of the model. Independent risk factor was screened by univariate and multivariate COX regression analysis. Spearman correlation was used to test the correlation between gene expression and stemness score, stromal score, immune score, and estimate score. Comparison of gene expression between the normal and tumors were performed in 18 cancer types which had more than five associated adjacent normal samples using linear mixed effects models. Mann–Whitney *U* test was used to compare the ssGSEA scores of immune cells or pathways between the high-risk and low-risk group.

## Results

### Identification of Ferroptosis-Related Long Non-coding RNA in Lung Adenocarcinoma Patients

**FIGURE 1:**
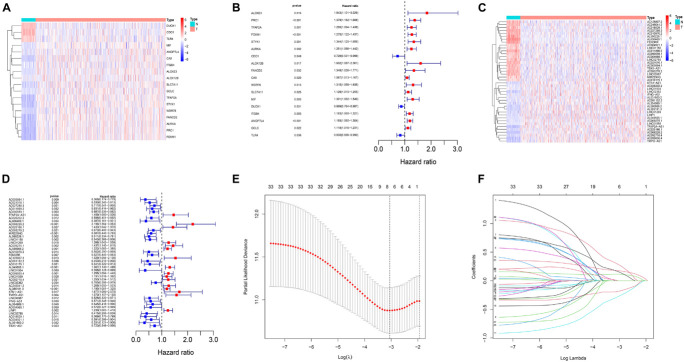
Identification of ferroptosis-related lncRNAs in LUAD patients and construction of a prognostic signature. **(A)** Heatmap for 18 differentially expressed genes, with red indicating high expression and blue indicating low expression. **(B)** Forest map for 18 prognostic genes. **(C)** Heatmap for 33 differentially expressed lncRNAs, with red indicating high expression and blue indicating low expression. **(D)** Forest map for 33 prognostic lncRNAs. **(E)** The optimal log λ value is indicated by the vertical black line in the plot. **(F)** The LASSO coefficient profile of ferroptosis-related lncRNAs, each line represents an independent lncRNA.

The workflow for construction of risk model and subsequent analyses is shown in Supplementary Figure 1. Firstly, a total of 283 ferroptosis-related gene expression matrices were extracted from the TCGA dataset. Among 283 genes, 18 genes were differentially expressed between tumor and adjacent normal tissues (Figure 1A) and consistent with univariate Cox regression analysis results (Figure 1B). A lncRNA whose expression value was correlated with one or more of the 18 ferroptosis-related genes was defined as a ferroptosis-related lncRNA. Through Pearson correlation analysis (| Pearson R| > 0.4 and *p* < 0.001), 590 ferroptosis-related lncRNAs were uncovered. Through analysis of differential expression and univariate Cox regression, we finally obtained 33 lncRNAs which were highly expressed in tumor and predicted a worse prognosis or were lowly expressed and predicted a better prognosis (Figures 1C,D).

### Construction and Validation of the Prognostic Signature for Ferroptosis-Related Long Non-coding RNA

**FIGURE 2:**
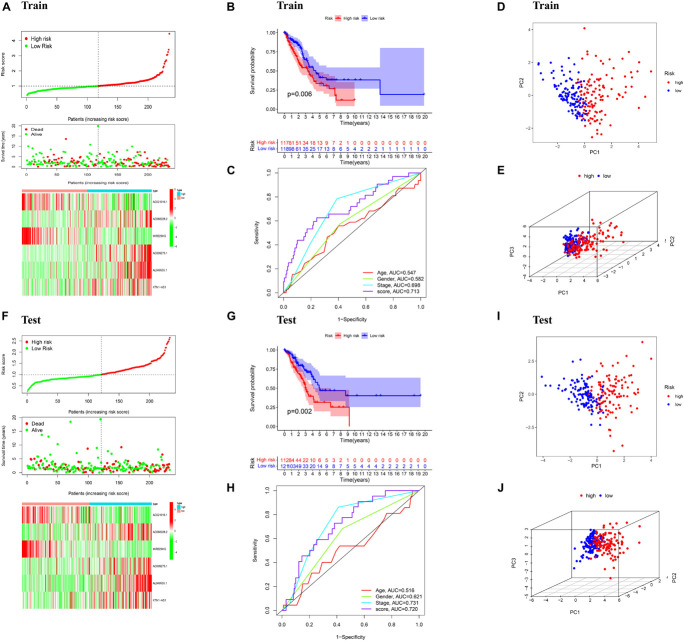
Validation of the prognostic signature. **(A)** Distribution of ferroptosis-related lncRNAs model based on risk score for the training set, patterns of the survival time, and survival status between the high- and low-risk groups for the training set and clustering analysis heatmap shows the display levels of the six ferroptosis-related lncRNAs for each patient in the training set. **(B)** Kaplan–Meier survival curves of the OS of patients in the high- and low-risk cohorts for the training set. **(C)** Time-dependent ROC analysis of accuracy of the model in the training set. **(D)** 2D and **(E)** 3D plots of the PCA of the training set. **(F)** Distribution of ferroptosis-related lncRNAs model based on risk score for the testing set, patterns of the survival time, and survival status between the high- and low-risk groups for the testing set and clustering analysis heatmap shows the display levels of the six ferroptosis-related lncRNAs for each patient in the testing set. **(G)** Kaplan–Meier survival curves of the OS of patients in the high- and low-risk cohorts for the testing set. **(H)** Time-dependent ROC analysis of accuracy of the model in testing set. **(I)** 2D and **(J)** 3D plots of the PCA of the testing set.

To build the ferroptosis-LPS for predicting the OS of LUAD patients, a total of 468 patients with LUAD were randomly classified into a training cohort (235 patients) and a test cohort (233 patients) at a 1:1 ratio firstly. Then we performed the least absolute shrinkage and selection operator (LASSO) regression analysis based on the expression values of 33 ferroptosis-related lncRNA in the training cohort (Figures 1E,F). Six lncRNAs, namely, AC021016.1, AC068228.2, MIR223HG, AC009275.1, AL049555.1, and KTN1-AS1, were identified. The risk scores of each patient in TCGA training and validation cohorts were calculated based on the coefficient for each lncRNA, and the formula is as follows: risk score = -0.1889 × AC021016.1 expression level) + (0.2885 × AC068228.2 expression level) - (0.2782 × MIR223HG expression level) + (0.1150 × AC009275.1 expression level) + (0.1759 × AL049555.1 expression level) + (0.1607 × KTN1-AS1 expression level). Patients in the TCGA training and test cohorts were divided into high-risk-score and low-risk-score subgroups based on the median value of risk scores. Risk score and survival status distributions are plotted in Figures 2A,F. The heatmap results suggested that AC021016.1 and MIR223HG were downregulated in the high-risk group, whereas the expression of AC068228.2, AC009275.1, AL049555.1, and KTN1-AS1 were upregulated in the high-risk group. Kaplan–Meier survival curves indicated that LUAD patients with low-risk scores had better clinical outcomes in either training or validation cohort (Figures 2B,G), and the ROC curves showed that ferroptosis-LPS had a promising ability to predict OS in the training and validation cohorts (Figures 2C,H). PCA analysis indicated the patients in two risk groups were distributed in two directions (Figures 2D,E,I,J).

### Independence of the Ferroptosis-LPS Considering Other Clinical Factors

**FIGURE 3:**
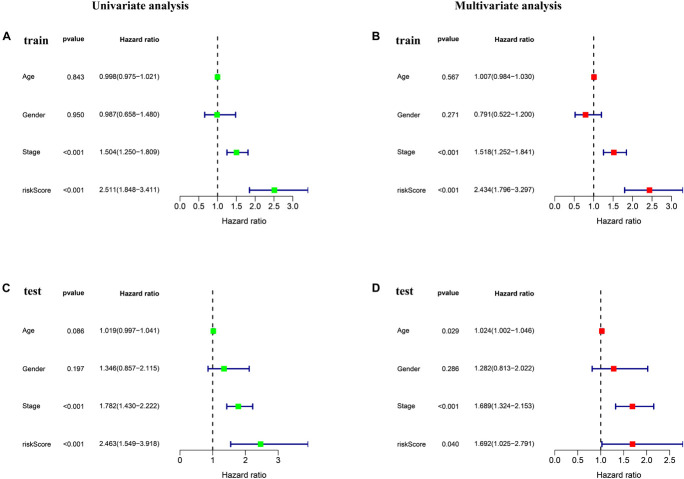
Ferroptosis-LPS was an independent prognostic factor for LUAD patients. **(A,B)** Univariate and multivariate Cox regression analyses in the training set. **(C,D)** Univariate and multivariate Cox regression analyses in the testing set.

Univariate and multivariate Cox regression were performed to assess whether ferroptosis-LPS was an independent prognostic factor for patients with LUAD. In the TCGA training dataset, univariate Cox analysis indicated that ferroptosis-LPS and stage were remarkably associated with OS (*p* < 0.001, Figure 3A) and multivariate Cox analysis further confirmed that ferroptosis-LPS was a prognostic risk factor (Figure 3B). The same result was found in the testing cohort, which confirmed that ferroptosis-LPS was an independent risk factor for LUAD patients (univariate: *p* < 0.001; multivariate: *p* < 0.05; Figures 3C,D). These results suggested that our ferroptosis-LPS might be useful for clinical prognosis evaluation.

### Functional Analyses in the TCGA Training and Testing Cohort

**FIGURE 4:**
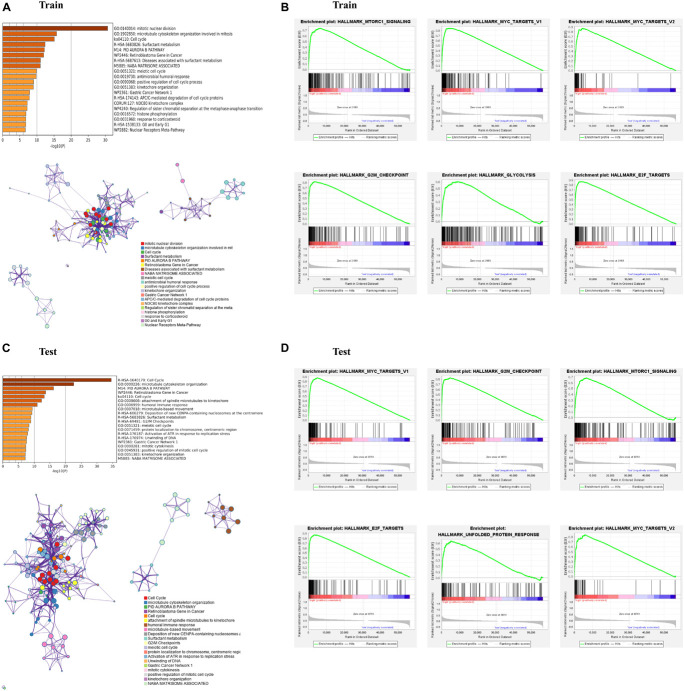
Enrichment analysis of differentially expressed genes (DEGs) between the high- and low-risk subgroups. **(A,C)** Bar graph of enriched terms across input gene lists, colored by *p*-values, and network of enriched terms, colored by cluster ID, where nodes that share the same cluster ID are typically close to each other. **(B,D)** Gene set enrichment analysis (GSEA) indicating that hallmarks were enriched in the high-risk subgroup of the training and testing sets. “Log10(P)” is the *p*-value in log base 10.

To investigate the biological functions and pathways which were associated with risk score in the training and test cohorts, the DEGs between the low- and high-risk groups were used to performed functional and pathway enrichment analysis. As expected, these DEGs were significantly enriched in cell cycle, retinoblastoma gene in cancer, and meiotic cell cycle (*p* < 0.05, Figures 4A,C). Gene set enrichment analysis showed that the genes in high-risk group of both train and test cohorts were significantly enriched in several hallmarks, such as MTORC1 signaling, MYC targets, G2M checkpoint, E2F targets, and so on (Figures 4B,D).

**FIGURE 5:**
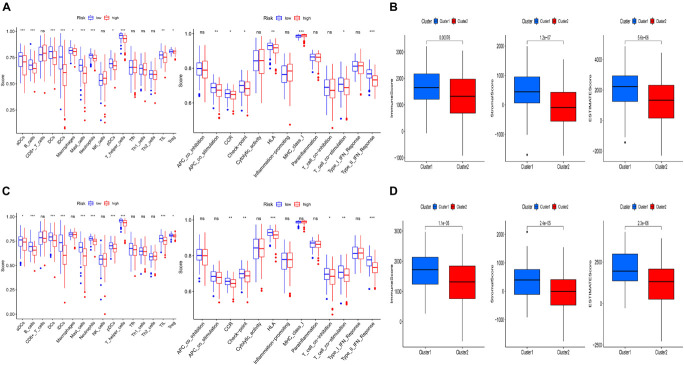
Comparison of immune status and tumor microenvironment between the high- and low-risk subgroups. **(A,C)** The scores of 16 immune cells and 13 immune-related functions of the training and testing set are displayed in boxplots. **(B,D)** The relationship between risk score with tumor microenvironment of the training and testing set. (cluster1: low-risk-score group; cluster2: high-risk-score group).

To further explore the relationship between the risk score and immune status, ssGSEA was performed to calculate the infiltrating score of immune cells and immune-related pathways. The scores of CCR, Check-point, HLA, T_cell_co-stimulation, and Type_II_IFN_Response were lower in the high-risk group, which were confirmed by the results of testing cohort. Moreover, the infiltrating scores of aDCs, B cells, DCs, iDCs, pDCs, neutrophils, T helper cells, and TIL were obviously higher in the low-risk groups of training and testing cohorts (Figures 5A,C). We further investigated the relationship between risk score with tumor microenvironment. Interestingly, the immune, stromal, and estimate score were higher in the low risk-score group (Figures 5B,D).

### Stratification Analysis of the Ferroptosis-LPS and Construction of the Ferroptosis-LPS-Based Nomogram

**FIGURE 6:**
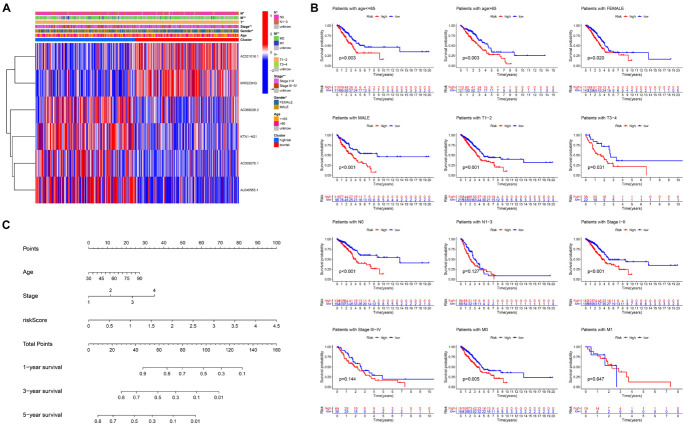
Stratification analysis of the ferroptosis-LPS and construction of the ferroptosis-LPS-based nomogram. **(A)** Heatmap of clinicopathological features and risk score of each patient in the entire TCGA dataset. **(B)** Survival analysis in various subgroups. **(C)** The nomogram predicts the probability of the 1-, 3-, and 5-year OS.

The clinicopathological features and risk score of each patient in the TCGA dataset are shown in the heatmap (Figure 6A). For better assessment of the prognostic ability of the FLPS, we performed a stratification analysis to confirm its ability to predict OS in various subgroups. Compared to low-risk groups, high-risk LUAD patients had worse OS in the male and female subgroup. The same results were found in patients with age ≤ 65 or > 65, T1-2 or T3-4, and stage I-II, N0 and M0 (Figure 6B).

To create a clinically applicable quantitative tool to predict the OS of LUAD patients, we established a nomogram using the risk score, age, and stage in the TCGA dataset (Figure 6C).

### Association of Six Ferroptosis-Related Long Non-coding RNAs With Tumor Microenvironment and Tumor Stemness

**FIGURE 7:**
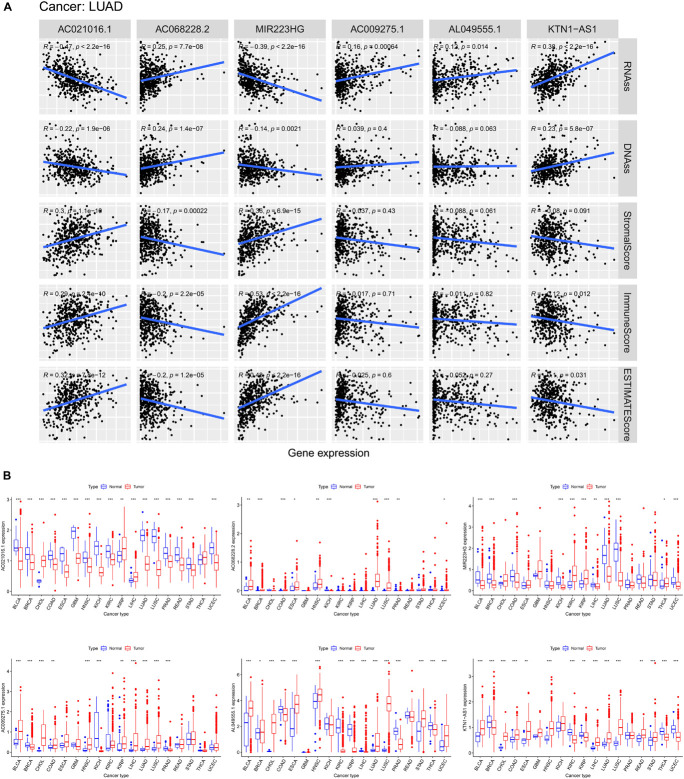
Association of expression of six lncRNAs with tumor microenvironment and tumor stemness and the pan-cancer expression analysis. **(A)** Correlation matrices between six lncRNAs expression and RNAss, DNAss, stromal score, immune score, and estimate score. **(B)** The expression level of six lncRNAs in tumors compared with normal tissue in 18 cancer types which were composed of more than five normal samples.

As the risk score was significantly correlated with tumor microenvironment, we investigated the association between the expression levels of the six ferroptosis-related lncRNAs and the tumor microenvironment. We found that AC021016.1 and MIR223HG had a positive correlation with stromal scores, immune score and estimate score, whereas AC068228.2 had a negative correlation (*p* < 0.001, Figure 7A).

In the process of cancer progression, tumor cells can gradually lose the phenotype of differentiation and acquire the characteristics of progenitor cells and stem cells, which might increase tumor resistance. Since those six lncRNAs were prognostic risk factors, we explored the correlation between them and RNA stemness score (RNAss) and DNA stemness score (DNAss), which were indicators of tumor stemness. The results showed that AC021016.1 and MIR223HG were negatively correlated with tumor stemness in both RNAss and DNAss while AC068228.2 and KTN1-AS1 had the positive correlation (*p* < 0.001, Figure 7A).

### Analysis of Six Ferroptosis-Related Long Non-coding RNAs in Pan-Cancer

To further understand the roles of six ferroptosis-related lncRNAs in tumors, we examined their expression levels in 18 cancer types in TCGA pan-cancer data. A striking inter-tumor heterogeneity of expression of six ferroptosis-related lncRNAs was observed. For instance, the expression levels of AC021016.1 showed the largest inter-tumor heterogeneity with some tumors having high levels of AC021016.1 (CHOL, KIRP, LIHC), while others were characterized with low levels of AC021016.1 expression (BLCA, BRCA, COAD, ESCA, GBM, HNSC, KICH, KIRC, LUAD, LUSC, PRAD, READ, STAD, UCEC). In addition, except MIR223HG, other ferroptosis-related lncRNAs had high expression levels in most tumor groups (Figure 7B).

Then we further explored the prognostic value of lncRNAs in tumors. Median was used to distinguish between high and low expression groups, whereas KM curves were performed to compare outcomes between the two groups. The results showed that the altered expression of ferroptosis-related lncRNAs were generally associated with patients’ overall survival. In most tumors, high expression of AC009275.1 and AC068228.2 were associated with poor prognosis, while other lncRNAs’ prognostic effect depended on the type of tumors (Supplementary Figure 2).

### Construction of the Competing Endogenous RNA Network and Functional Enrichment Analysis

**FIGURE 8:**
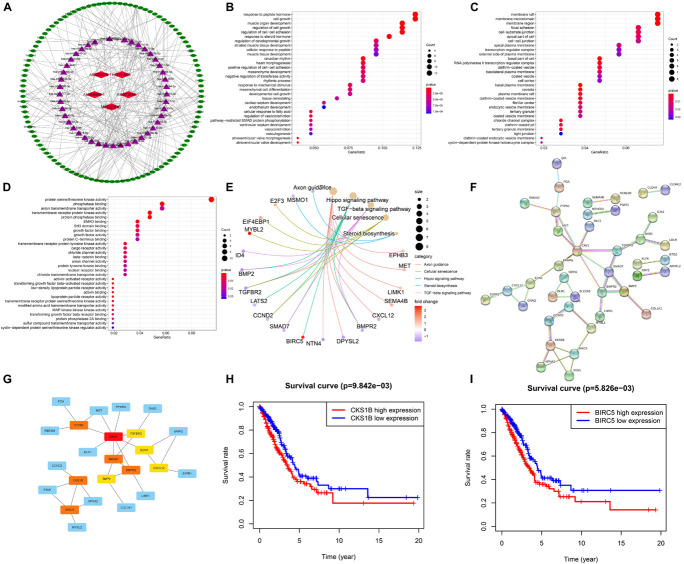
Construction of the ceRNA network and screening of hub genes. **(A)** The ceRNA Network which consists of five ferroptosis-related lncRNAs, 45 miRNAs and 107 mRNAs. **(B–D)** Bubble plots showing GO analysis of 107 genes for biological process, cellular component and molecular function. **(E)** Cnetplot for KEGG signal pathway of 107 genes. **(F,G)** The PPI network of 41 genes and hub genes among them. **(H,I)** Kaplan–Meier survival curves of BIRC5 and CKS1B.

Several lncRNAs were found to act as miRNA sponges to participate in the regulation of gene expression. Then we explored the DEMs and DEGs based on the TCGA-LUAD dataset (|log2FC| > 1, FDR < 0.05); 369 DEMs and 1,517 DEGs were identified by the “edgeR” package. Top 20 up- and downregulated DEMs and DEGs are shown in Supplementary Figure 3. Using the LncBase Predicted v.2, we predicted the targeted miRNA of the six ferroptosis-related lncRNAs with a 0.6 threshold. Then the intersection of target miRNAs for downregulated lncRNAs with upregulated miRNAs of TCGA dataset and target miRNAs for upregulated lncRNAs with downregulated miRNAs of LUAD were taken. To further explore the target genes of the miRNAs, we applied TargetScan, miRDB, and miRWalk to predict the target genes. We used the negative correlation criteria [(a) miRNAs should be targeted to the mRNA; (b) the expression level of mRNAs should be opposite to miRNAs; and (c) the mRNAs belong to DEGs] to screen the downstream mRNAs. Ultimately, five ferroptosis-related lncRNAs, 45 miRNAs, and 107 mRNAs were included in our ceRNA network (Figure 8A).

Furthermore, Go and KEGG enrichment analyses were performed to clarify the biological function of 107 downstream genes. Through BP analysis, response to peptide hormone, cell growth, regulation of cell growth, and regulation of cell-cell adhesion were obviously enriched (Figure 8B). The results of CC analysis contained membrane raft, membrane microdomain, membrane region, and focal adhesion (Figure 8C). Moreover, MF analysis revealed that downstream genes were mostly enriched in protein serine/threonine kinase activity (Figure 8D). KEGG analysis demonstrated that axon guidance, hippo signaling pathway, TGF-beta signaling pathway, cellular senescence, and steroid biosynthesis were the top five enriched pathways (Figure 8E).

### Screening of Hub Genes and Survival-Related Genes

Through STRING database, a total of 41 genes were screened out of 107 target genes to construct the PPI network, which contained 41 nodes and 42 edges (Figure 8F). The 10 hub genes (CAV1, BIRC5, CKS1B, SMAD7, BMPR2, PTPN1, BMP2, EDN1, TGFBR2, and CXCL12) were screened out by the rank of degree which was calculated by cytoHubba in cytoscape (Figure 8G). The KM curve showed that the expression of BIRC5 and CKS1B were negatively correlated with survival prognosis (*p* < 0.01, Figures 8H,I).

### Validation of the Expression Level of Ferroptosis-Related Long Non-coding RNAs in Lung Adenocarcinoma Samples

To validate the expression levels of ferroptosis-related lncRNAs, we detected two ferroptosis-related lncRNAs expression levels in 20 LUAD samples and 20 adjacent normal tissues through qRT-PCR. The results showed that AC021016.1 was significantly downregulated in LUAD while KTN1-AS1 was highly expressed in LUAD (Supplementary Figure 4).

## Discussion

Long non-coding RNAs (lncRNAs) are a kind of non-protein coding RNAs with over 200 nucleotides (Wang J. et al., 2021). They are widely expressed in human cells and play vital roles in regulation of pathological and physiological processes in various types of malignant tumors (Zhou et al., 2020). Previous studies have constructed a few of lncRNA prognosis signatures, like m6A-related lncRNA signature (Xu et al., 2021), immune-related lncRNA signature (Wang J. et al., 2021), and autophagy-related lncRNA signature (Zhou et al., 2020), which can be used to identify high-risk patients and judge the prognosis of them. Ferroptosis is a form of regulatory cell death and plays an important role in the occurrence and development of LUAD. Some studies have shown that lncRNAs can participate in the regulation of ferroptosis and growth of LUAD (Sánchez et al., 2014; Johnson et al., 2017; Zhang et al., 2017; Wang et al., 2019; Gai et al., 2020). Thus, ferroptosis-related lncRNAs are potential markers for prognosis and a prognostic model based on them for LUAD is still lacking.

To construct a prognostic model, we firstly uncovered 18 ferroptosis-related genes through gene expression differential analysis and Cox regression. Among them, cysteine dioxygenase 1 (CDO1), toll-like receptor 4 (TLR4), and dual oxidase 1 (DUOX1) were downregulated in tumors and played a protective role, while Fanconi anemia complementation group D2 (FANCD2) and others were with high expression in tumors and were risk factors for prognosis. CDO1 was found to transform cysteine to taurine while cysteine was an indispensable substrate of glutathione peroxidase 4 (GPX4), a lipid repair enzyme of ferroptosis. The suppression of CDO1 increased the expression of GSH and inhibited ferroptosis in gastric cancer (Hao et al., 2017). In another study (Chen et al., 2019), Chen et al. reported that the silence of TLR4 and NOX4 significantly retarded the autophagy and ferroptosis in rats with heart failure. FANCD2, a nuclear protein that responds to DNA damage repair, negatively regulated ferroptosis of bone marrow injury in cancer treatment and could be a potential target of anticancer therapies (Song et al., 2016). In addition, a 10 ferroptosis-related genes signature and a ferroptosis-related gene signature with five genes were constructed to predict the prognosis of patients with LUAD (Wang S. et al., 2021; Zhu et al., 2021). In general, ferroptosis-related genes play important roles in the progression of LUAD.

It has been reported that ferroptosis-related lncRNAs were prognostic risk factors and the signatures constructed with ferroptosis-related lncRNAs can be used to distinguish high-risk patients from low-risk patients (Cai et al., 2021; Tang et al., 2021). However, the full role of ferroptosis-related lncRNAs in LUAD remains to be further explored. Here, we mined ferroptosis-related lncRNAs through Pearson correlation and constructed a prognostic Signature with six ferroptosis-related lncRNAs. Through Kaplan–Meier survival curves, ROC curve, PCA, and univariate and multivariate COX regression, we further confirmed the validity of our signature. Among the six lncRNAs, AC021016.1 and MIR223HG were downregulated in LUAD and were protective factors for prognosis, while KTN1-AS1 and others were upregulated and were risk factors for prognosis. LncRNA KTN1-AS1 was upregulated in several tumors, such as non-small cell lung cancer, bladder cancer, head and neck squamous cell carcinoma, and hepatocellular carcinoma and promoted the progression of tumors (Zhang et al., 2019; Jiang et al., 2020; Hu et al., 2021; Li Y. Y. et al., 2021). In our study, we find that lncRNA KTN1-AS1 was significantly upregulated in LUAD and correlated with tumor stemness and tumor microenvironment. In another study (Geng et al., 2021), MIR223HG was found to be a genome instability-related lncRNA and used to construct a genome instability-related lncRNA signature, which further confirmed the potential function of MIR223HG in LUAD. AL049555.1 was demonstrated to be highly expressed and associated with high risk of PAAD (Wang et al., 2020). The function of AC021016.1, AC068228.2, and AC009275.1 has not been elucidated and our research uncovered their potential roles in LUAD.

To further elucidate the potential function of six ferroptosis-related lncRNAs, we constructed ceRNA network to find the downstream genes. Through STRING and Cytoscape, we found the 10 hub genes of PPI network. Among them, the high expression of BIRC5 and CKS1B were significantly correlated with poor prognosis. BIRC5 is a well-known therapeutic target of cancers (Li et al., 2019) and plays an important role in cell division and inhibits cell death (Wheatley and Altieri, 2019). Previous research revealed that BIRC5 was elevated and promoted the development of lung cancer (Han et al., 2020). CKS1B, a member of Cks/Suc1 family, is involved in the regulation of cell cycle and chemotherapeutic resistance in cancers (Wang et al., 2017). In lung cancer, it also promotes cell growth and induces drug resistance (Shi et al., 2020). In addition, although CAV1, SMAD7, BMP2, EDN1, and CXCL12 were not significantly different in the prognostic analysis in our study, they also play important roles in the occurrence and development of lung cancer (Katsura et al., 2018; Yan et al., 2019; Huang et al., 2020; Pulido et al., 2020; Tong et al., 2020). In general, the functions of target genes further confirmed the potential roles of those lncRNAs. Finally, we detected two ferroptosis-related lncRNAs expression levels through qRT-PCR with 20 pairs of samples, which increased the reliability of our results.

Ferroptosis is a new form of cell death that might potentially provide a new strategy in clinical cancer therapy. However, the crosstalk among ferroptosis and other cell death processes, auto-immune microenvironment, and maintenance and transformation of tumor stem cells remain unsolved. In this study, we constructed a ferroptosis-related lncRNA signature and confirmed its validity in predicting survival outcomes of LUAD. We hope that these findings will provide some useful insights for clinical practice. However, several limitations were in our study. Firstly, multicenter LUAD datasets are needed to verify the validity of our model. Secondly, although we confirmed the expression in our own samples through qRT-PCR, the application of this prognostic prediction model in LUAD need to be further explored.

## Data Availability Statement

The original contributions presented in the study are included in the article/Supplementary Material, further inquiries can be directed to the corresponding author/s.

## Ethics Statement

The studies involving human participants were reviewed and approved by the Medical Ethics Committee of Changhai Hospital, Navy Military Medical University. The patients/participants provided their written informed consent to participate in this study.

## Author Contributions

XF, CH, and XW constructed this study. XF, CH, XW, CJL, and HC collected and analyzed the data. BS and CL wrote and revised the manuscript and were responsible for the critical reading of the manuscript. All authors contributed to the article and approved the submitted version.

## Conflict of Interest

The authors declare that the research was conducted in the absence of any commercial or financial relationships that could be construed as a potential conflict of interest.

## Publisher’s Note

All claims expressed in this article are solely those of the authors and do not necessarily represent those of their affiliated organizations, or those of the publisher, the editors and the reviewers. Any product that may be evaluated in this article, or claim that may be made by its manufacturer, is not guaranteed or endorsed by the publisher.

## Footnotes

1
https://portal.gdc.cancer.gov/

2
https://xenabrowser.net/datapages/

3
http://carolina.imis.athena-innovation.gr

4
http://www.targetscan.org

5
http://www.mirdb.org/miRDB/

6
http://mirwalk.umm.uni-heidelberg.de/

7
https://string-db.org/
