# Supplementary figures and images for: Construction of a Ferroptosis-Related Long Non-coding RNA Prognostic Signature and Competing Endogenous RNA Network in Lung Adenocarcinoma
Source: Front Cell Dev Biol. 2021 Nov 8;9:751490. doi: 10.3389/fcell.2021.751490 (PMC8606539; doi:10.3389/fcell.2021.751490)

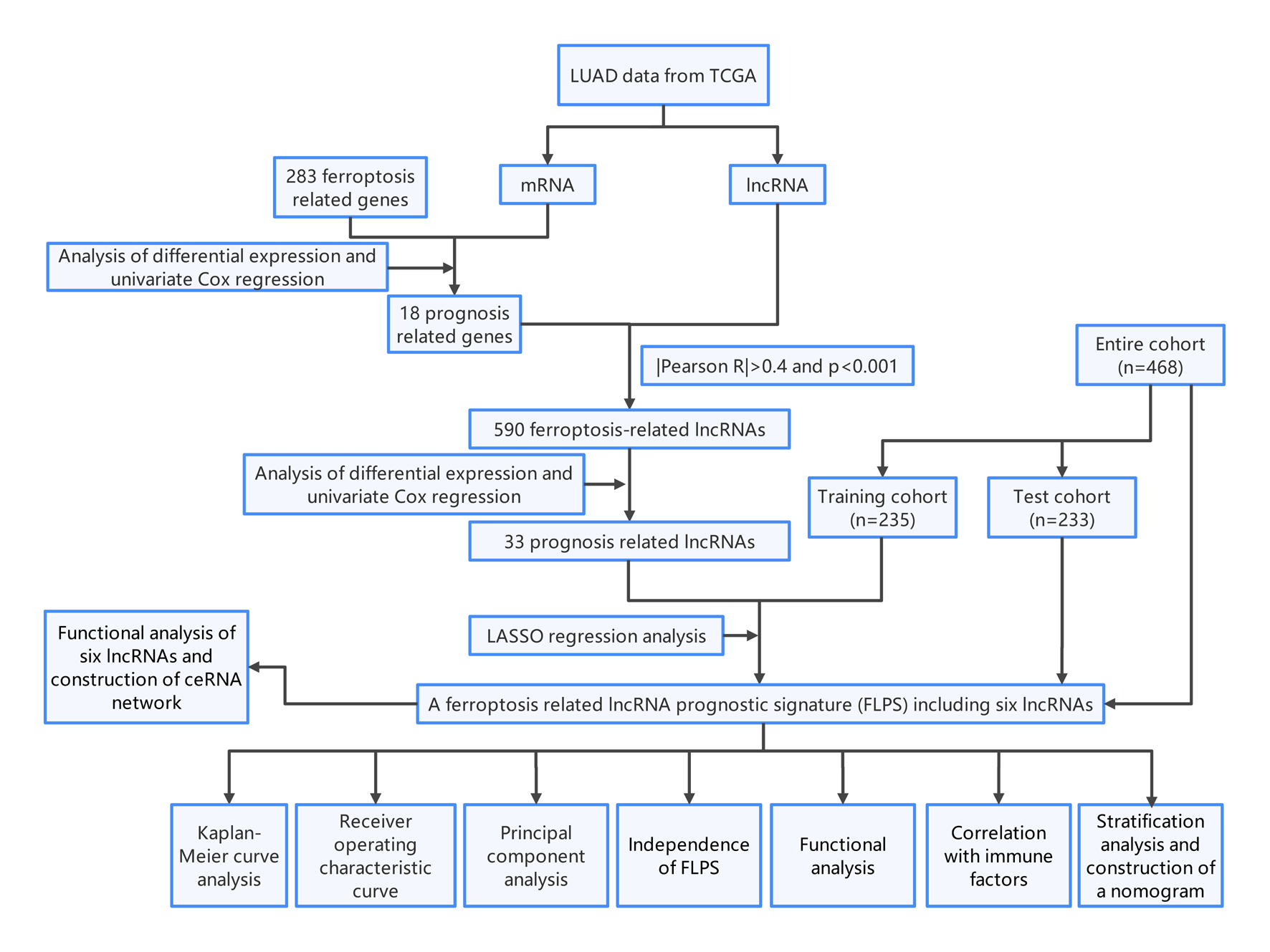

Supplement: Supplementary file 1 [file Image_1.JPEG]

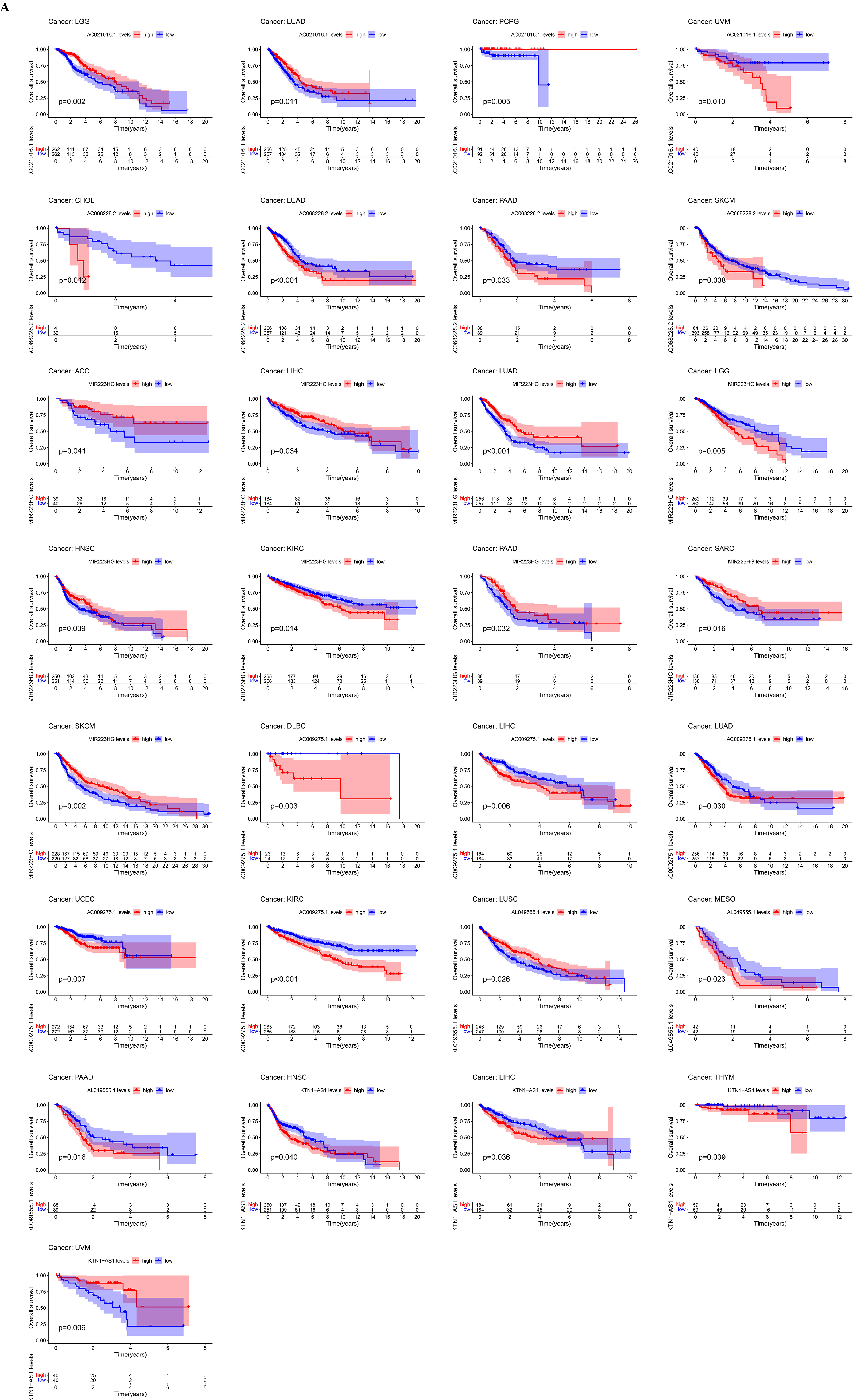

Supplement: Supplementary file 2 [file Image_2.JPEG]

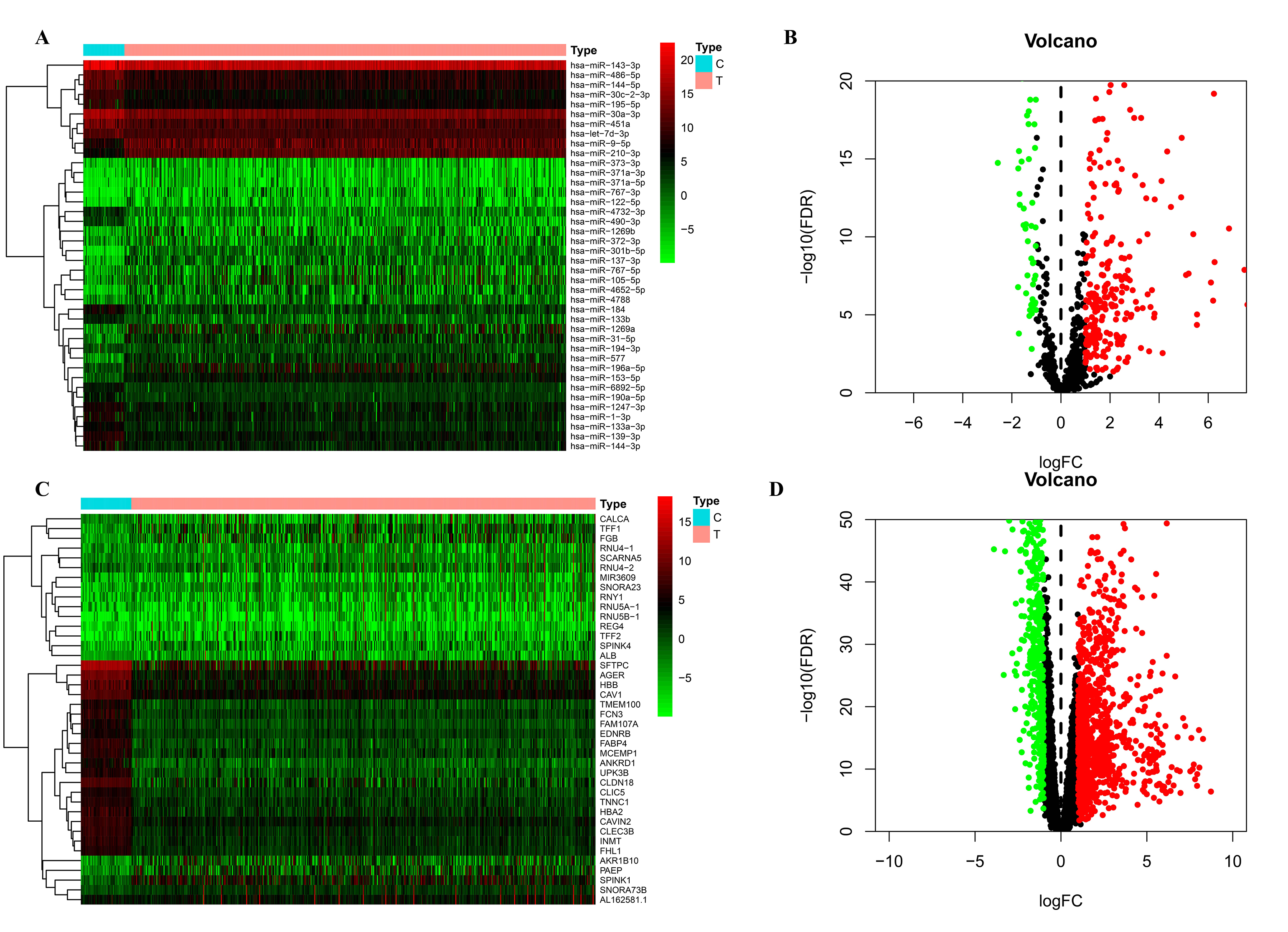

Supplement: Supplementary file 3 [file Image_3.JPEG]

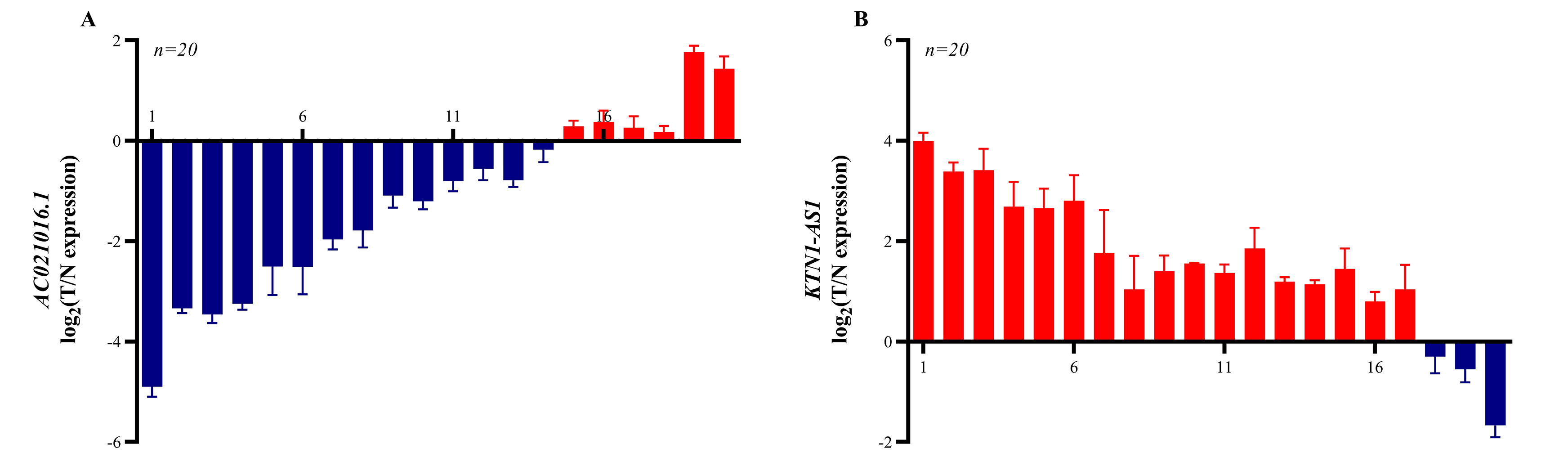

Supplement: Supplementary file 4 [file Image_4.JPEG]
